# Vanillin Inhibits Translation and Induces Messenger Ribonucleoprotein (mRNP) Granule Formation in *Saccharomyces cerevisiae*: Application and Validation of High-Content, Image-Based Profiling

**DOI:** 10.1371/journal.pone.0061748

**Published:** 2013-04-24

**Authors:** Aya Iwaki, Shinsuke Ohnuki, Yohei Suga, Shingo Izawa, Yoshikazu Ohya

**Affiliations:** 1 The Department of Applied Biology, Graduate School of Science and Technology, Kyoto Institute of Technology, Matsugasaki, Kyoto, Japan; 2 The Department of Integrated Biosciences, Graduate School of Frontier Sciences, University of Tokyo, Kashiwa, Chiba, Japan; Univ. of Edinburgh, United Kingdom

## Abstract

Vanillin, generated by acid hydrolysis of lignocellulose, acts as a potent inhibitor of the growth of the yeast *Saccharomyces cerevisiae*. Here, we investigated the cellular processes affected by vanillin using high-content, image-based profiling. Among 4,718 non-essential yeast deletion mutants, the morphology of those defective in the large ribosomal subunit showed significant similarity to that of vanillin-treated cells. The defects in these mutants were clustered in three domains of the ribosome: the mRNA tunnel entrance, exit and backbone required for small subunit attachment. To confirm that vanillin inhibited ribosomal function, we assessed polysome and messenger ribonucleoprotein granule formation after treatment with vanillin. Analysis of polysome profiles showed disassembly of the polysomes in the presence of vanillin. Processing bodies and stress granules, which are composed of non-translating mRNAs and various proteins, were formed after treatment with vanillin. These results suggest that vanillin represses translation in yeast cells.

## Introduction

Lignocellulosic materials, such as stove residues and wood chips, are potential substrates for bioethanol production [1], [2]. At present, these materials have not been put into practical use, partly because the by-products of acid hydrolysis of lignocellulose inhibit yeast growth and fermentation. Vanillin is one of the major phenolic compounds produced by degradation of lignin, one of the components of lignocellulose. Since vanillin is a more potent inhibitor of fermentation than other by-products [3], it is considered a harmful compound produced by pretreatment of lignocellulosic biomass.

Vanillin is also one of the most important flavor compounds. Most of the world’s vanillin is synthesized from petrochemicals or wood pulp lignins. *De novo* synthesis of vanillin was implemented in *Saccharomyces cerevisiae*, with the aim of offering a sustainable alternative means of vanillin production [4]. Since vanillin is toxic to yeast, breeding of vanillin-tolerant yeast strains is an important prerequisite for a viable vanillin cell factory [5].

Despite much interest in the inhibitory effects of vanillin, little is known about its intracellular target and its mode of action in *S. cerevisiae* [6]. Genome-wide, fitness-based screening identified 76 vanillin-sensitive mutants [7]. The non-essential deletion mutants exhibiting vanillin-sensitivity were classified into the functional categories “chromatin remodeling”, “vesicle transport” and “ergosterol biosynthetic process”, suggesting that these functions are important for vanillin tolerance. Another screening identified various vanillin-tolerant strains [8], [9]. A high-ergosterol-containing strain was more tolerant to vanillin, suggesting that high ergosterol content was responsible for vanillin tolerance [8]. These studies indicated that various intracellular processes affect vanillin sensitivity, although the mechanism of vanillin tolerance remains obscure.

In addition to the fitness-based approach, a compendium approach that involves assessment of multiple cellular response parameters can be used to infer the drug targets of a chemical [10]. Recently, we developed an image-based profiling method to infer drug targets in *S. cerevisiae* based on the morphological changes induced by the drug [11]. After quantification of the morphology of the wild-type yeast cells treated with the drug, its morphological profile was compared with those induced by a deletion of non-essential 4,718 genes by calculating the correlation coefficients. Based on the similarity in each mutant, we were able to infer intracellular targets ranging the 4,718 non-essential genes.

In this study, we applied high-content, image-based profiling to predict the intracellular processes affected by vanillin in *S. cerevisiae.* The prediction was verified by examining polysome reduction and messenger ribonucleoprotein (mRNP) granule formation [12]–[14] after treatment of yeast cells with vanillin. Our results indicate that vanillin inhibits translation and induces mRNP granule formation.

## Materials and Methods

### Yeast Strains, Chemicals and Growth Conditions

The strains used in this study are shown in Table S1. Cells were grown in YPD medium containing 1% Bacto Yeast Extract (Becton, Dickinson and Company, USA), 2% polypeptone (Becton, Dickinson and Company) and 2% glucose (Nacalaitesque, Japan), with or without vanillin (Kanto Chemicals, Japan) in the presence of 0.1% DMSO, at 25°C. For the analyses of polysome profiles and mRNP granules, cells were grown in SD medium containing 0.67% Yeast Nitrogen Base w/o Amino Acids (Becton, Dickinson and Company) and 2% glucose with appropriate amino acids and bases. Stock solutions of 2 M vanillin were prepared in DMSO (Wako Pure Chemical Industries, Japan) and stored at −20°C. Samples for analysis with CalMorph were taken in the log-phase of growth (4×10^6^–1×10^7^ cells/ml) except for time-course experiments. For the time-course experiments, Δ*his3* Δ*adh6* cells were inoculated into YPD medium containing 4 mM vanillin and incubated at 25°C. Cells were fixed at 0, 1, 2, 4, 6, and 8 h after inoculation. Five replicated experiments for each time point were done. For dose-response experiments, cells were inoculated into YPD medium containing 0, 0.25, 0.5, 0.75, and 1 mM vanillin and incubated at 25°C until concentrations of cells reached to 4×10^6^–1×10^7^ cells/ml. Four replicated experiments for each vanillin concentration were done.

### Fluorescence Staining and Microscopy

Cells were fixed for 30 min in growth medium supplemented with formaldehyde (final concentration; 3.7%) and potassium phosphate buffer (100 mM, pH 6.5) at 25°C. Cells were collected by centrifugation and further incubated in potassium phosphate buffer containing 4% formaldehyde for 45 min at room temperature. Actin staining was performed by overnight treatment with 15 U/ml Rhodamine-phalloidin (Invitrogen Corp, USA) and 1% Triton-X in PBS. Then, the cells were mixed with 1 mg/ml FITC-conjugated concanavalin A (Sigma, St. Louis, MO, USA) in P buffer (10 mM sodium phosphate and 150 mM NaCl, pH 7.2) for 10 min to stain mannoprotein on the cell surface. After washing twice with P buffer, the cells were mixed with mounting buffer (1 mg/ml *p*-phenylenediamine, 25 mM NaOH, 10% PBS and 90% glycerol) containing 20 µg/ml DAPI (Sigma) for DNA staining. The specimens were observed with Axioimager, equipped with a ×100 ECplan-Neofluar lens (Carl Zeiss, Germany), a CoolSNAP HQ cooled-CCD camera (Roper Scientific Photometrics, Tucson, AZ, USA) and Axio Vision software (Carl Zeiss).

### Image Processing

Image-processing was performed with CalMorph (ver.1.2). CalMorph is a program that can obtain a large amount of data regarding many morphological parameters of individual cells, including cell cycle phase, cell forms, *etc*., from a set of pictures of cell walls, nuclei and actins. The CalMorph user manual is available at the *S. cerevisiae* morphological database (SCMD) [15] (http://scmd.gi.k.u-tokyo.ac.jp/datamine/).

### Statistical Analysis

The Jonckheere–Terpstra test, principal component analysis (PCA), Pearson product-moment correlation analysis and bootstrap-based estimation of the false discovery rate (FDR) were implemented as described previously [11]. All statistical analyses were performed using R (http://www.r-project.org/).

**Figure 1:**
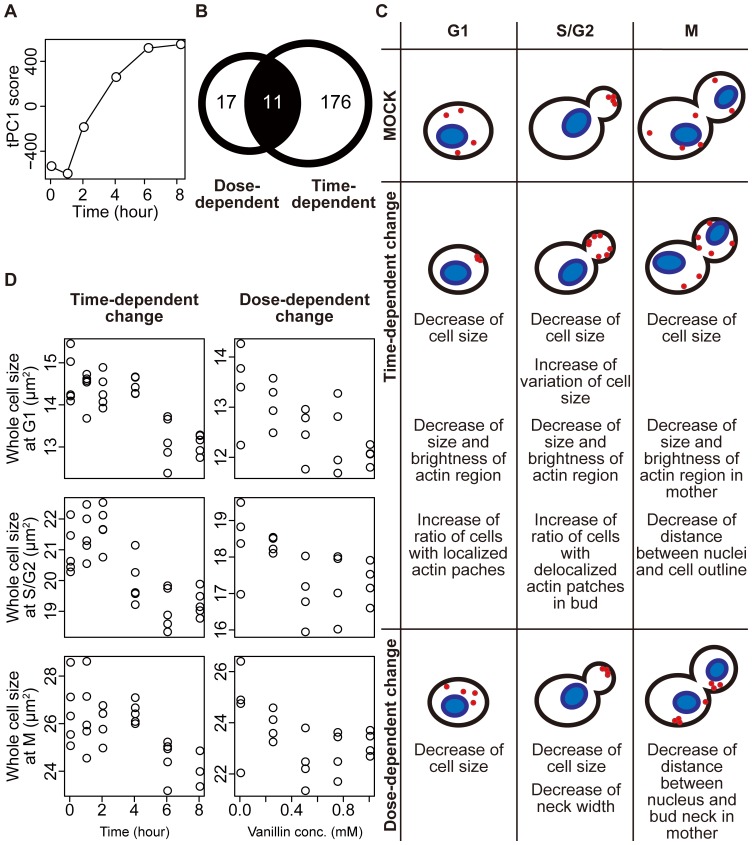
Morphological changes of yeast (*Saccharomyces cerevisiae*) cells after treatment with vanillin. (A) Time-dependent change in the tPC1 score. Wild-type (Δ*his3* Δ*adh6*) cells were pre-cultured in YPD medium, inoculated into YPD medium containing 4 mM vanillin at 1×10^6^ cells/ml and incubated at 25°C. The cells were fixed at the indicated time points and stained with fluorescent dyes. Then, at least 200 cells were photographed under the fluorescence microscope and analyzed with CalMorph. For each parameter, the parameter values at each time point were summarized into one value from five replicate experiments with rank-order (see Materials and Methods) and subjected to PCA. The contribution ratio of tPC1 was 0.559. (B) Venn diagram of dose-dependent and time-dependent parameters after vanillin treatment. For dose-dependency, wild-type cells were grown in YPD medium containing 0.00, 0.25, 0.50, 0.75 and 1.00 mM vanillin. For time dependency, time-course data were acquired as described in (A). Of the 501 parameters, 28 and 187 were dose- and time-dependent, respectively, at *P*<0.01. The permutation tests determined that less than four and approximately four parameters were expected to be detected by chance at this *P* value, respectively. The parameters significantly changed in same direction (increase or decrease) were counted as the parameters changed similarly in both experiments. (C) Illustration of representative wild-type cells after vanillin treatment. In each dataset, the successive PCA [16] was performed to illustrate the representative cell shape. Blue filled ellipses and red circles indicate nuclei and actin patches, respectively. (D) Decrease in cell size after vanillin treatment. The parameter values of C11-1_A, C101_A1B and C101_C in each dataset were plotted for whole cell size in G1, S/G2 and M, respectively. For a full definition of the parameters, see [17].

To summarize the features of the cell morphology changed by the vanillin treatment, we applied the successive PCA on the morphological data obtained from the time-course and the dose-response experiments of Δ*his3* Δ*adh6* cells, as described previously [16]. The replicated sample values obtained from the image analysis of time-course experiments were combined together, ranked among the combined samples, and summed into one rank-sum value under each time-point. To standardize the rank-sum values among the parameters, the rank-sum values of cells at 0 h after the vanillin treatment were subtracted from the rank-sum values of all samples in each parameter. Then, the standardized rank-sum values were subjected to PCA (first PCA). Similarly, values from the dose-response experiments were combined, rank-summed, standardized with 0 mM vanillin treatment samples, and subjected to first PCA. The principal component (PC) from the PCA on the time-course data and the dose-response data were referred to as tPC and dPC, respectively, hereafter. The tPC1 and the dPC1 that explained a large part (46.8% and 55.9%, respectively) of the variance of the rank-sum values showed time- and dose-dependent change, respectively (Fig. 1A and data not shown). To detect parameters contributing to tPC1 and dPC1, the PC loadings were calculated from the correlation coefficients between the rank-sum values and the PC scores. By the permutation test of 1000 iterations for the loadings, the false discovery rate (FDR) was determined at an arbitrary threshold. At FDR = 0.1, 105 and 17 of 501 parameters were detected to have significant loadings for the tPC1 and dPC1, respectively. To determine the morphological features accompanied by the tPC1 of the first PCA, a second PCA was performed for the 105 significantly contributing parameters to tPC1 using morphological data from the 122 replicated experiments of Δ*his3* as a null distribution. The PCs of the second PCA were named in alphabetical order (e.g. PC1, PC2, and PC3 were named tPC1a, tPC1b, and tPC1c, respectively). For the tPC1, first eight PCs (tPC1a-tPC1h) explained 62.7% of the variance, and had significant loadings for 51, 38, 26, 13, 13, 7, 7 and 6 of 105 parameters at P<0.05 after the Bonferroni correction. For the dPC1, dPC1a explained 75.0% of the variance, and had significant loadings for 13 of the 17 parameters at P<0.05 after the Bonferroni correction. Based on these detected parameters, the features of the cell morphology changed by the vanillin treatment in each experiment were described in Fig. 1C.

To evaluate the similarities between morphologic changes in vanillin-treated, Δ*his3* Δ*adh6* cells *versus* systematic deletion mutant strains, the morphological profile of Δ*his3* Δ*adh6* cells treated with various concentrations of vanillin was calculated as follows. First, for every morphological parameter, values of all vanillin concentrations and replicates were summarized into a Z score from the Jonckheere–Terpstra test. Each Z score represents the dose-dependency of the parameter under a normal distribution. Then, to reduce dimensions, the resultant Z scores were projected to 21 PCs that were obtained with PCA on null distributed data (n = 122). The cumulative contribution ratio (CCR) of the 21 PCs was reached to 70%. These PC scores obtained from the Z scores represent the profile of morphologic changes that resulted from treatment with vanillin. On the other hand, the 21 PC scores of 4718 nonessential deletion mutants were calculated from data of SCMD [17] as described previously [11]. Finally, the correlation coefficient R and the associated *P* value for the 21 PC scores from above-calculated vanillin-treated, wild-type cells and that from every deletion mutant strain were calculated to evaluate the similarities between morphologic changes in vanillin-treated, wild-type cells *versus* mutant strains.

To compare the morphological profile of vanillin-treated Δ*his3* and Δ*erg6* cells (Fig. S2), cells were cultured in YPD at 25°C with 0, 0.25, 0.5, 0.75, and 1 mM of vanillin and morphological quantification was done as described above. For every morphological parameter, values from all five replicates and vanillin concentrations were summarized into a Z score from the Jonckheere–Terpstra test. To reduce dimensions, the resultant 501 Z scores were projected to 102 PCs that were obtained from PCA on null distributed data (n = 122), where the CCR of the 102 PCs was reached to 99%. The calculated 102 PC scores of Δ*his3* and Δ*erg6* cells were plotted.

### Gene Ontology Terms

The gene ontology annotations used in this study were obtained from the “GO Term Finder” (http://www.yeastgenome.org/cgi-bin/GO/goTermFinder.pl) in the *Saccharomyces* Genome Database (SGD) website. Query genes were obtained from the analyzed CalMorph data, and the background gene set was 4,708 genes (out of 4,718 non-essential genes) that were associated with at least one GO term.

### Alcohol Dehydrogenase (ADH)6 Gene Deletion

PCR was performed using the ExTaq Kit (TaKaRa, Shiga, Japan). The plasmid used as template was pYO2241 (pBS-CgLEU2). A set of primers (Forward 5′-CATTCGAGGAAGAAATTCAACACAACAACAAGAAAAGCCAAAATCTCGAGGTCGACGGTATC-3′, and Reverse 5′-AGCAGTTAAAAAGAAAGGAGCTACATTTATCAAGAGCTTGACAACCGCTCTAGAACTAGTGGATC-3′) was used to amplify the *Candida glabrata LEU2* gene sequence in pYO2241 and 45 base pairs up- and down-stream of the sequence of the *ADH6* open reading frame. The amplified PCR products were purified by ethanol-precipitation and introduced into yeast cells (Δ*his3*) [18]. Transformants were selected in SD medium lacking leucine. Deletion of the *ADH6* gene was confirmed by PCR using a set of primers (Forward 5′-CAATTCAATCTAATTTAATA-3′, Reverse 5′-TATATCGAT TAAAACAGCAC-3′).

### Measurement of Vanillin Concentration in Yeast Culture

YPD medium containing vanillin was inoculated with yeast cells. At each time interval during culture, 100-µl samples were removed, centrifuged (13000×g for 3 min) and the supernatants were collected. After 50-fold dilution in 100 mM Tris buffer (pH 7.8), vanillin levels were measured spectrophotometrically at 340 nm. The concentration in the supernatants was calculated using the absorptivity of vanillin that was established experimentally [19].

### Polysome Profile Analysis

BY4741 cells in SD medium were taken during the log-phase of growth (*ca*. 1×10^7^ cells/ml). Preparation of the yeast extract and sucrose gradient for separation of the polysome fraction was carried out using a gradient master and fractionator (107–201 M and 152-002, BioComp Instruments, Canada) by the method of Inada and Aiba [20].

### Observation of mRNP Granule Formation

BY4741 cells expressing GFP-fusion proteins in the log phase of growth were further incubated with various vanillin concentrations. Vanillin-treated cells were observed immediately without fixation using a Leica AF6500 fluorescence microscope. The plasmids used for the observation of cytoplasmic processing bodies (P-bodies) and stress granules (SGs) were described previously [21]–[23]. In order to count the number of GFP foci in the entire cell cytoplasm, six images were captured in a Z-stack. One hundred cells under each condition were examined and the experiments were repeated 3 times (300 cells in total were examined). The data are represented as means ± SD.

## Results

### Morphological Changes Induced by Vanillin

Vanillin is mainly degraded to vanillyl alcohol in *S. cerevisiae* [19]. Because this reaction is catalyzed by Adh6 and Adh7 *in vitro* [24], [25], we compared the vanillin bioconversion in Δ*his3* (*ADH6 ADH7*), Δ*adh6* (*ADH7*) and Δ*adh7* (*ADH6*) cells. The yeast cells were cultured in YPD containing 2.5 mM vanillin and incubated at 25°C for 24 h. Vanillin breakdown occurred in the cultures of Δ*his3* and Δ*adh7* cells but not Δ*adh6* cells (Fig. S1), suggesting that this bioconversion was dependent on Adh6. Therefore, in subsequent morphologic profiling experiments, we used YOC4538 (*adh6*-deleted Δ*his3* strain) as wild-type to avoid artifacts due to long-term exposure to metabolites of vanillin and their effects on yeast cell morphology.

Yeast cell morphology was visualized by fluorescence microscopy after staining for cell shape, actin and nuclear DNA. The images were processed using CalMorph to obtain values for 501 morphologic parameters. Time-dependent morphological changes in wild-type cells were analyzed by PCA after treatment with 4 mM vanillin. PCA projects multi-dimensional phenotypic data into the two major orthogonal factors that capture the most variance, making possible visualization of the time-dependent dynamics of the morphological changes in the resulting two-dimensional space. Fig. 1A shows that after a short lag period, the tPC1 value increased in a time-dependent manner up to 8 h.

Dose-dependent morphological changes were examined using a lower concentration of vanillin to minimize non-specific effects. Addition of 1 mM vanillin reduced the growth rate by only 10%; therefore, the wild-type cells were treated with 0, 0.25, 0.50, 0.75 and 1.00 mM vanillin and incubated at 25°C for 16 h to ensure the maximal morphological changes. The Jonckheere–Terpstra test indicated that 28 of the 501 parameter values exhibited significant dose-dependent changes over this concentration range, with *P*<0.01 (Table S2). The dose-dependent parameters were then compared with the 187 time-dependent parameters whose values were changed after a 6-h incubation with 4 mM vanillin at *P*<0.01 (Table S2). As illustrated by a Venn diagram (Fig. 1B), 11 of the 28 dose-dependent parameter values were changed after 6 h incubation. This implies that the values in the rest (17 parameters) changed after prolonged incubation for maximal change. Fig. 1C illustrates some of the time- and dose-dependent morphological changes that occurred in typical cells. One of the dose-dependent parameters was “whole cell size”. Fig 1D shows that the size of vanillin-treated cells at every stage of the cell cycle was reduced in a dose-dependent manner. Vanillin-treated cells did not arrest at any specific cell cycle stage.

### Yeast Mutants Morphologically Similar to Vanillin Treated Cells

To identify nonessential deletion mutants with similar morphology to vanillin-treated cells, we used an inference algorithm to estimate similarities between induced morphological changes [11]. Briefly, mutants that were morphologically similar to vanillin-treated cells were statistically identified using the following four steps: 1) morphological analysis of wild-type cells using CalMorph after treatment with 0, 0.25, 0.50, 0.75 or 1.00 mM vanillin at 25°C for 16 h; 2) PCA of dose-dependent morphological changes based on the eigenvector calculated from Δ*his3* data to avoid any correlation among the parameters; 3) similar PCA of the 4,718 nonessential deletion mutants [17]; and 4) evaluation of the similarities between vanillin-treated cells and the 4,718 mutant cells by calculating the Pearson product-moment correlation coefficient (R) and the associated *P* value for the principal component scores.

**Figure 2:**
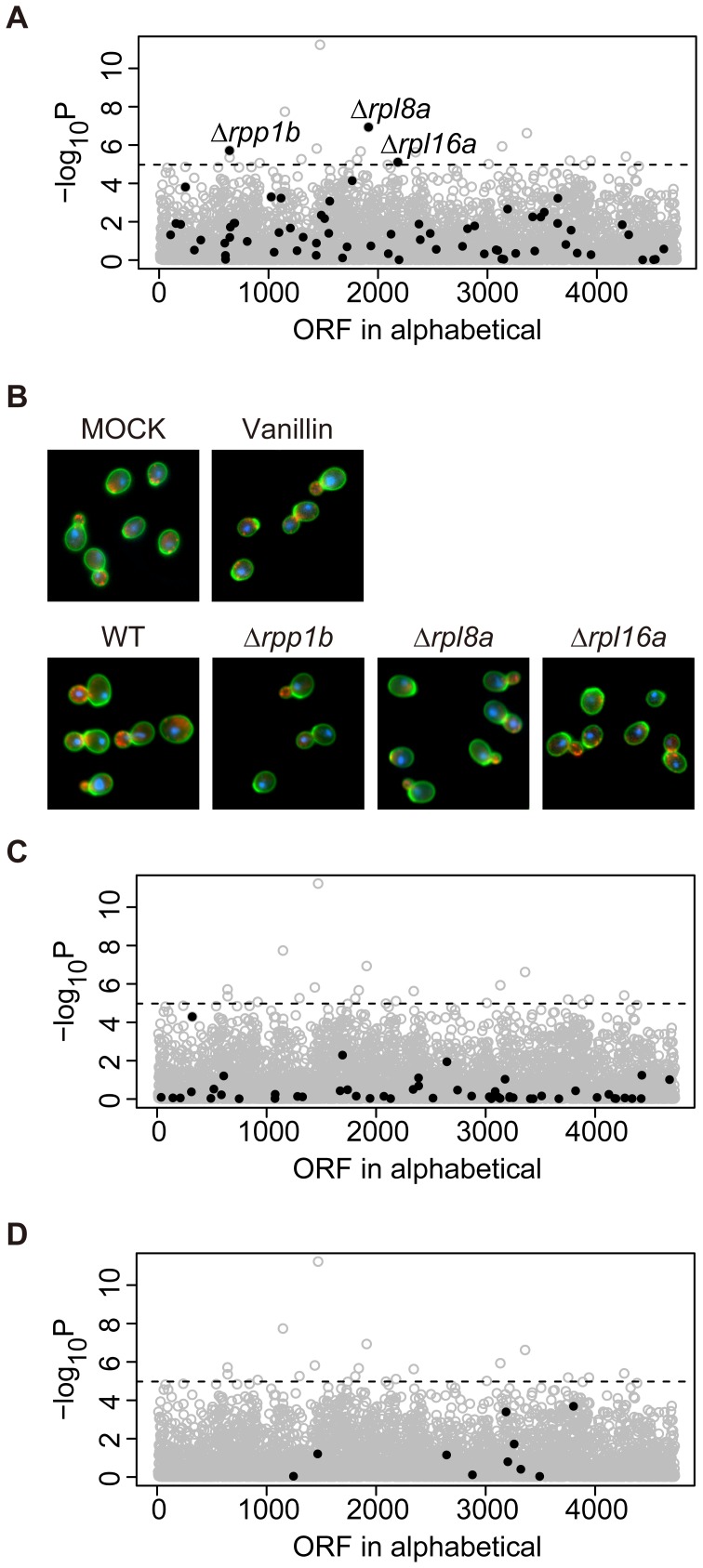
Distribution of morphological similarity between vanillin-treated cells and non-essential deletion mutants. Morphological data of wild-type cells incubated with various vanillin concentrations were used to infer the cellular processes affected by vanillin, as described previously [11]. The X-axis shows the ORFs in alphabetical order. The Y-axis indicates –log_10_ P of the correlation coefficient (R) between morphological profiles of vanillin-treated cells and that of each of the 4,718 non-essential gene deletion mutants (grey circles). The horizontal dashed line indicates a P value of 0.05 after Bonferroni correction. (A) Distribution of the cytosolic large ribosomal subunit (GO:0022625) mutants (black filled circles). (B) Photographs of Δ*his3* Δ*adh6* cells treated with 0.1% DMSO (MOCK), Δ*his3* Δ*adh6* cells treated with 1 mM vanillin (Vanillin), Δ*his3* (WT), and the significantly similar deletion mutants of the large ribosomal subunit. Cells were stained with FITC-ConA (green) for cell wall, Rh-ph (red) for actin and DAPI (blue) for DNA. (C) Distribution of the cytosolic small ribosomal subunit (GO:0022627) mutants (black filled circles). (D) Distribution of the ergosterol biosynthetic pathway (GO:0006696) mutants (black filled circles).

Among the 4,718 mutants, 18 morphologic profiles were significantly similar to that of the vanillin-treated cells at a one-sided *P*<0.05 with Bonferroni correction. Table S3 shows the significantly similar mutants and their molecular functions. Of the 18 top-ranked mutants, three, Δ*rpl8a*, Δ*rpp1b* and Δ*rpl16a* (Figs. 2A and 2B), were categorized as belonging to a “cytosolic protein component of the 60S (large) ribosomal subunit” (GOID: 0022625) in the gene ontology (GO) database. This GO term had the most significant relative occurrence (*P* = 0.0695), as revealed by GO term finder [26]. The 60S ribosomal mutants were 11.7-fold enriched in the significantly similar mutants (16.7%, 3 out of the 18 mutants) compared to the all nonessential deletion mutants (1.4%, 67 out of the 4708 mutants). None of the mutants defective in the cytosolic protein component of the 40S (small) ribosomal subunit exhibited phenotypes that were similar to vanillin-treated cells (Fig. 2C).

**Figure 3:**
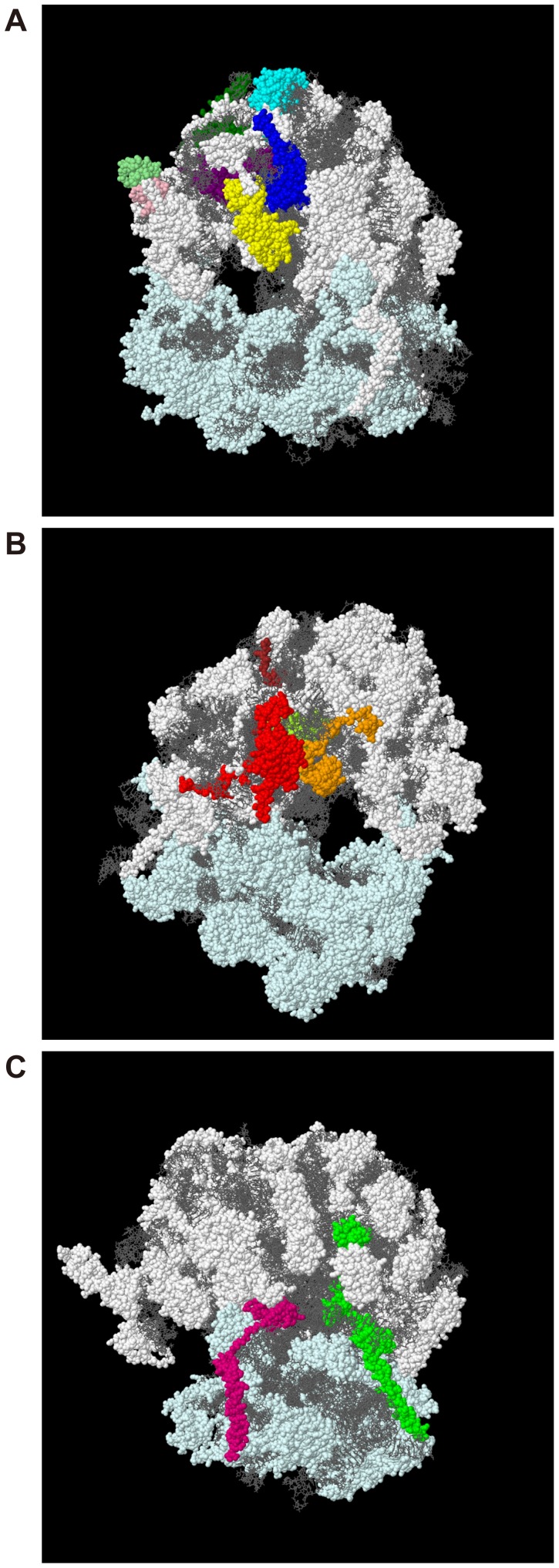
High-ranked components of the large ribosomal subunit (R>0.50) in the X-ray structure of the yeast ribosome. The X-ray crystal structure of the yeast 80S ribosome [27] was used to map the 15 large ribosomal subunit components whose deletion mutants had a>0.5 correlation coefficient with vanillin-treated cells. White and light-cyan spheres indicate the large (60S) and small (40S) subunits, respectively. The gray wire-frame indicates rRNA. (A) Cluster of subunits close to the mRNA tunnel entrance. Cyan, dark green, yellow, blue, purple, pink and light green spheres indicate Rpl6b, Rpl7a, Rpl9a, Rpl16a/b, Rpl20a, Rpp1b and Rpp2b, respectively. (B) Cluster of subunits close to the mRNA tunnel exit. Red, green yellow, orange and brown spheres indicate Rpl8a, Rpl15b, Rpl36a and Rpl37b, respectively. (C) Cluster of subunits at the backbone. Green and magenta spheres indicate Rpl19a and Rpl24a/b, respectively.

We mapped 15 high-ranked components of the large ribosomal subunit (R>0.50) against the X-ray structure of the yeast ribosome [27]. As shown in Fig. 3, these components were localized to three domains. Rpp1b, Rpp2b, Rpl6b, Rpl7a, Rpl9a, Rpl16a/b and Rpl20a were located in the tunnel entrance of mRNA, including the stalk structure. Rpl8a, Rpl15b, Rpl36a and Rpl37b were located in the tunnel exit. Rpl19a and Rpl24a/b were located in the backbone with functioning linkage to the small subunit. Thus, the deletion mutants that exhibited phenotypes similar to the vanillin-treated cells were all missing components that mapped to three distinct domains of the large ribosomal subunit.

### Morphological Changes of Mutants of Ergosterol Biosynthesis

A previous genome-wide screening identified 76 vanillin-sensitive mutants [7]. Among them were several deficient in ergosterol biosynthesis (Δ*erg2*, Δ*erg3*, Δ*erg6*, and Δ*erg24*). The reported cross-sensitivity of these ergosterol biosynthetic mutants to other compounds generated from lignin, including furan derivatives, weak acids, and phenolic compounds, suggested that ergosterol is a key component of tolerance to various inhibitors. To determine whether vanillin affects ergosterol biosynthesis itself, we compared the morphological phenotypes of the *erg* mutants with those of the vanillin-treated cells. Fig. 2D shows that no nonessential *erg* mutants exhibited phenotypes similar to the vanillin-treated cells, implying that reduction of ergosterol biosynthesis and treatment with vanillin have different effects on cell morphology. We also compared the morphological changes induced by vanillin in one *erg* mutant (Δ*erg6*) from those induced in Δ*his3* *ERG6*). The morphological features induced by vanillin in Δ*erg6* were essentially the same as those of vanillin-treated Δ*his3* cells (Fig. S2). This indicates that the effects of vanillin are the same in both ergosterol-reduced and -sufficient conditions, suggesting that the reduction in ergosterol and the effects of vanillin are unrelated. Taken together, these results lead us to conclude that ergosterol biosynthesis is not a direct target of vanillin.

### Polyribosome Reduction by Vanillin Treatment

**Figure 4:**
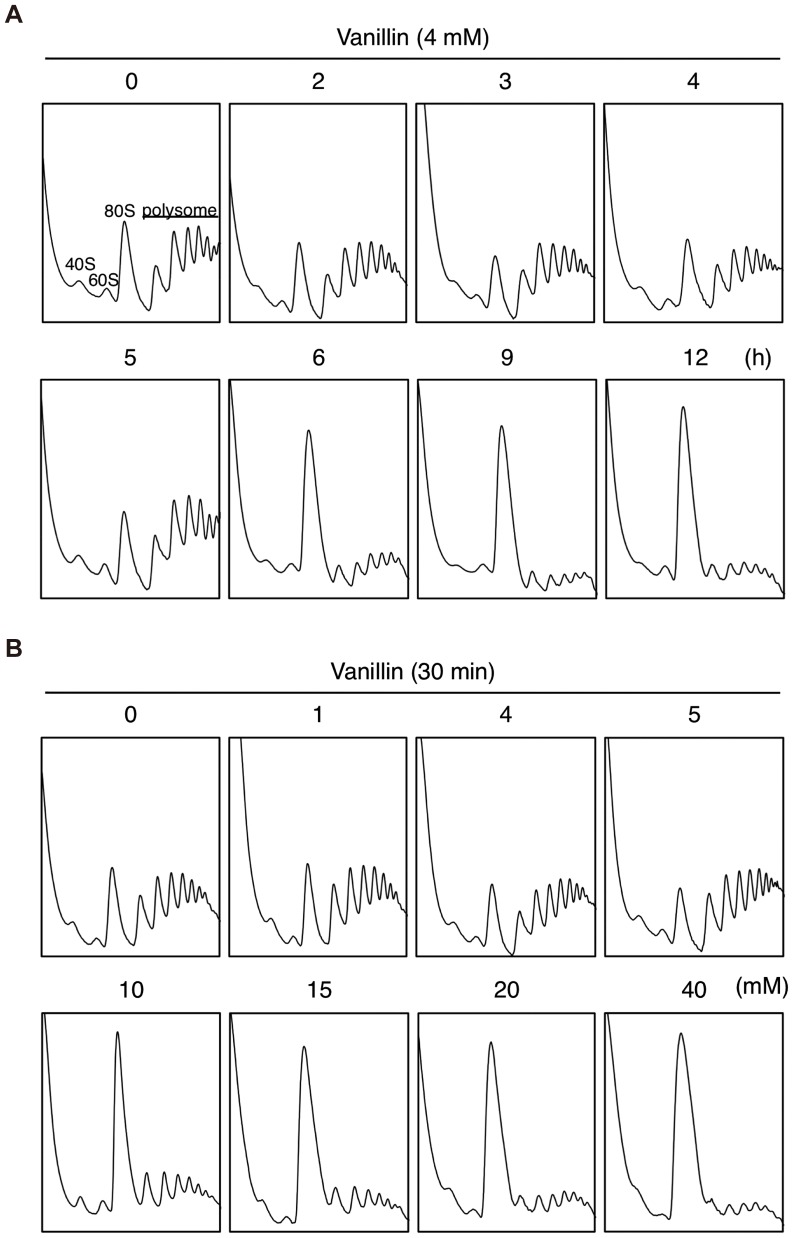
Polysome profile analysis of cells after treatment with vanillin. BY4741 cells at a concentration of 1×10^6^ cells/ml in SD medium were treated with 4 mM vanillin for 0–12 h (A) or 0–40 mM vanillin for 30 min (B). The polysome, 40S (small ribosomal subunit), 60S (large ribosomal subunit), and 80S (monosome) peaks are labeled.

Since we found morphological similarities between vanillin-treated cells and large ribosomal subunit mutants, we evaluated polysome profiles to determine whether vanillin affects bulk translation activity. Treatment with 4 mM vanillin for 6 h induced apparent morphological changes and reduced translation activity (Figs. 1A and 4A). We also observed that incubation of cells with 4 mM vanillin for a longer period (9–12 h) led to a drastic reduction in the polysome fraction, with a concomitant increase in the monosome fraction (80S) (Fig. 4A), suggesting that 4 mM vanillin caused an attenuation of bulk translation activity.

We also assessed translation activity after 30 min incubation with vanillin (Fig. 4B). Although less than 5 mM vanillin failed to induce pronounced reduction in the polysome fraction, treatment with over 10 mM vanillin clearly reduced the polysome fraction, and the 80S monosome fraction was increased by treatment with 10 mM or more vanillin. Since rapid reduction in the polysome fraction is correlated with a reduction in translation initiation [28], these results suggest that treatment of yeast cells with vanillin causes repression of bulk translation activity.

### Vanillin Induces Formation of P-bodies and SGs

**Figure 5:**
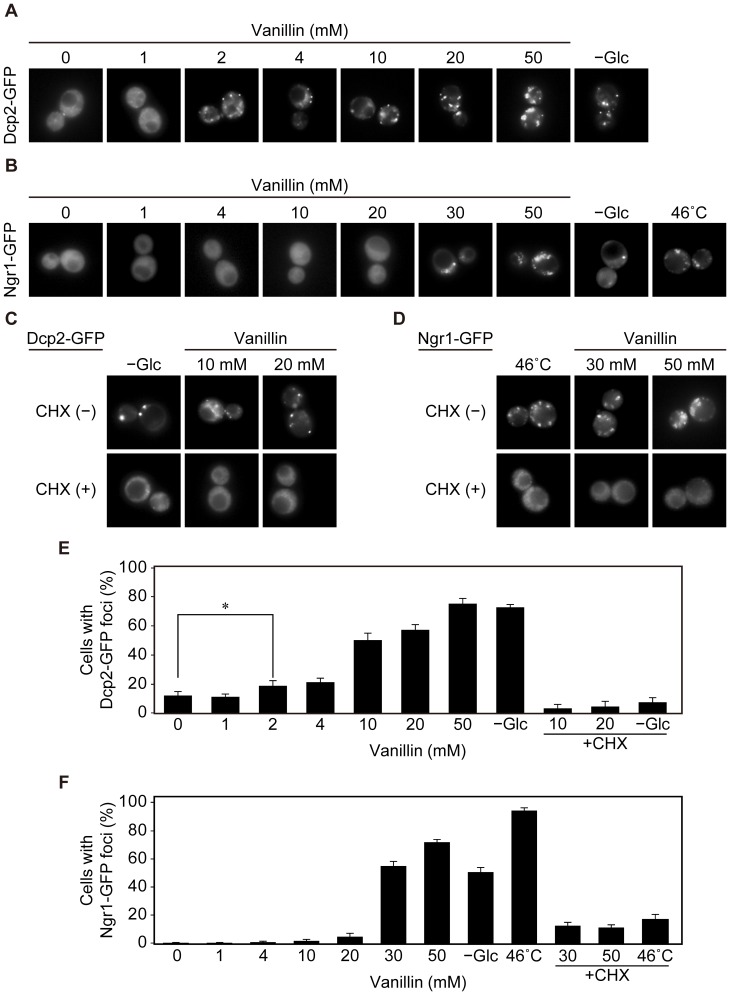
Formation of cytoplasmic processing bodies (P-bodies) and stress granules (SGs) after treatment with vanillin. (A and B) BY4741 cells expressing indicated GFP-fusion constructs at a concentration of 1×10^6^ cells/ml in SD medium were treated with vanillin (0–50mM) for 30 min, deprived of glucose (- Glc) for 15 min, or administered heat shock at 46°C for 10 min. The assembly of P-bodies and SGs was examined with Dcp2-GFP (A) and Ngr1-GFP (B), respectively. (C and D) Cells were treated with cycloheximide (CHX) (100 µg/ml) for 1 min prior to treatment with vanillin (0–50 mM) for 30 min, glucose deprivation (- Glc) for 15 min, or robust heat shock at 46°C for 10 min. The assembly of P-bodies and SGs was examined using Dcp2-GFP (C) and Ngr1-GFP (D), respectively. (E and F) Percentages of cells containing cytoplasmic granule(s) of Dcp2-GFP (E) or Ngr1-GFP (F). The data are represented as means ± SD. Asterisk indicates P<0.05.

Attenuation of bulk translation activity is often associated with the accumulation of non-translating mRNAs into cytoplasmic messenger ribonucleoprotein (mRNP) granules, including P-bodies and SGs [29]. Therefore, we investigated the effects of vanillin (1–50 mM) on the formation of P-bodies and SGs in yeast cells. Dcp2-GFP and Ngr1-GFP were used as markers of P-bodies and SGs, respectively [14], [29]. Like glucose depletion, vanillin enhanced the formation of clear foci of Dcp2-GFP in the cytoplasm after 30 min incubation (Fig. 5A). The minimum concentration of vanillin that resulted in generation of Dcp2-GFP foci was 2 mM (Figs. 5A and 5E). The number of Dcp2 cytoplasmic granules of was greater after treatment with 50 mM vanillin (Fig. 5A). These results indicate that >2 mM vanillin induced the formation of P-bodies. Like glucose depletion and robust heat shock at 46°C, the formation of SGs was induced by vanillin (Fig. 5B). In this case, however, more than 30 mM vanillin was required to form clear foci of Ngr1-GFP in the cytoplasm (Figs. 5B and 5F).

Cycloheximide (CHX) prevents the assembly of P-bodies and SGs by trapping mRNAs in polysomes [29], [30]. Therefore, we evaluated the effects of CHX on the vanillin-induced formation of P-bodies and SGs. Cells were treated with CHX (100 µg/ml) for 1 min prior to treatment with vanillin, and then the assembly of P-bodies and SGs was investigated. Consistent with previous reports, pre-treatment with CHX prevented the formation of Dcp2-GFP and Ngr1-GFP foci upon glucose deprivation and robust heat shock at 46°C, respectively (Figs. 5C–5F) [29]. Likewise, CHX prevented the formation of Dcp2-GFP and Ngr1-GFP foci caused by vanillin (Figs. 5C–5F), indicating that vanillin caused the formation of P-bodies and SGs via the release of non-translating mRNAs from polysomes.

We verified that vanillin caused the formation of P-bodies using other markers. Dhh1, Pat1, Xrn1, Lsm1, Scd6, Edc3, and Dcp1 are core components of P-bodies in *S. cerevisiae* [14]. Like glucose depletion, >10 mM vanillin induced clear cytoplasmic foci as revealed by these markers (Fig. S3). Therefore, we conclude that vanillin induces the formation of P-bodies.

We also investigated the localization of other SG components by using Pab1-GFP, Pbp1-GFP, Hrp1-GFP, Rpg1-GFP, Prt1-GFP, and Nip1-GFP as markers [12], [30]. Heat shock induces SGs, as revealed using all of the above markers (Fig. S4). Conversely, glucose depletion enhanced only the formation of clear foci of Pab1-GFP, Pbp1-GFP, and Hrp1-GFP, but not Rpg1-GFP, Prt1-GFP, and Nip1-GFP in the cytoplasm. Fig. S4 shows that >30 mM vanillin induced clear cytoplasmic foci, as revealed by Pab1-GFP, Pbp1-GFP, Hrp1-GFP but not by Rpg1-GFP, Prt1-GFP, and Nip1-GFP (Fig. S4). Based on these results, we conclude that the effects of vanillin on the induction of SG formation are similar to those of glucose depletion.

## Discussion

This study addressed the intracellular mode of action of vanillin in *S. cerevisiae.* We demonstrated that vanillin inhibits translation, causes the accumulation of cytoplasmic mRNP granules and induces morphological phenotypes similar to those induced by mutations in the cytosolic protein component of the large ribosomal subunit. Since translation is a fundamental process and is required for cell proliferation, these findings help explain the toxic effects of lignocellulose-derived inhibitors, of which vanillin is the most potent [6]. It seems probable that the compounds produced by acid hydrolysis of lignocellulose inhibit yeast growth and fermentation by reducing translation. Previous studies have suggested that ergosterol biosynthesis is a key component of vanillin tolerance. However, our phenotypic analyses indicate that ergosterol biosynthesis is not a direct target of vanillin.

We used high-content, image-based profiling to predict the intracellular targets of vanillin. Ohnuki *et al*. [11] reported a proof-of-concept study of four well-characterized bioactive compounds that provided reasonable assurance of the efficacy of the method. The aim of the current study was to predict the target of a compound whose target was unknown. We chose vanillin for three reasons. First, its molecular mode of action in yeast is of interest to many researchers because of its industrial value and potential implications for energy policy. Second, it is well known that vanillin inhibits yeast growth and ethanol production, but its intracellular target is unknown. Third, preliminary studies revealed that vanillin induces morphological changes in yeast cells. The current method allows the systematic prediction of drug targets based on the morphological phenotypes of nonessential gene deletion mutants, without any advance knowledge of the compound. This contrasts with other conventional high-content imaging approaches, which focus on specific bioactivities (*e*.*g*., translocation of fluorescently labeled cellular targets between intracellular compartments) to assign the drug candidate to well-characterized compound groups [31], [32].

Translational repression induces the formation of cytoplasmic mRNP granules, such as P-bodies and SGs. In contrast, P-bodies contain proteins involved in translational repression (IF4ET, Rck/p54/Dhh1, CPEB1, and the RISC complex), suggesting that their formation represses translation activity [33]. Therefore, it is of interest to investigate the cause-and-effect relationship between translational repression and formation of mRNP granules after treatment with vanillin. We suppose that suppression of translation might be the initial intracellular event. As previously reported [12]–[14], P-bodies increased in number and size when translation is repressed to accumulate free untranslated mRNA. We observed that none of the mutants defective in P-body and SG components had morphological phenotypes similar to those of vanillin-treated cells. In addition, none of those mutants show strong vanillin-sensitive phenotype [7]. Therefore, it is likely that accumulation of cytoplasmic mRNP granules is a consequence of translational repression induced by vanillin. An alternative possibility is that vanillin may initiate formation of P-bodies before translational repression. Vanillin has been shown to inhibit the repair of breaks in double-stranded DNA in human cells [34], and the replication inhibitor hydroxyurea induces the formation of P-bodies in yeast cells [35], suggesting that replication stress is a potent inducer of P-bodies. However, it is unlikely that vanillin affects DNA synthesis and/or DNA repair because 1) unlike hydroxyurea-treated cells, vanillin treatment did not arrest the cell cycle at S/G2, and 2) vanillin-treated cells did not exhibit morphological phenotypes that are characteristic of other DNA-replication or DNA-repair mutants [17]. Finally, it is also possible that vanillin affects the process of ribosome assembly. In this context, it should be noted that Rpp1 is also required for processing of precursor tRNA and rRNA [36].

The size of vanillin-treated cells was reduced in a dose-dependent manner. Likewise, many mutants defective in the large ribosomal subunit exhibit a small cell size [17], [37]. In contrast, other morphological traits specific to vanillin-treated cells were not always observed in the large ribosomal subunit mutants. Although Δ*rpl8a*, Δ*rpp1b*, and Δ*rpl16a* mutants exhibited phenotypes that were remarkably similar to vanillin-treated cells, other large ribosomal subunit mutants were not so similar. The less-expressed counterparts (Δ*rpl8b*, Δ*rpp1a*, and Δ*rpl16b*) did not exhibit similar phenotypes with vanillin-treated cells. The vanillin treatment possibly affected the activity of the large subunit, since no nonessential mutants of small ribosomal subunit exhibited similar phenotypes to vanillin-treated cells. The strains with phenotypes similar to vanillin-treated cells were deletion mutants, whose deleted components were locally enriched in the three domains on the surface of the ribosome. Strikingly, all large subunit mutants did not exhibit the same morphological phenotypes, perhaps due to the specialized function of each subunit gene. Another possibility is that some of the ribosomal mutants had weak morphological phenotypes. Among 67 non-essential mutants of the large ribosomal subunit, 30 were low-ranked (R <0.3) and exhibited phenotypes different from vanillin-treated cells. Among these dissimilar mutants, 19 (63%) exhibited mild morphological changes with differences in at least 1 of 501 traits [17]. Possibly due to overlapping and redundant functions of the subunit genes, the large ribosomal subunit deletion mutants with weak morphological phenotypes were not detected as similar mutants to vanillin-treated cells.

A previous study [7] identified 76 vanillin-sensitive mutants that were classified into the functional categories of “chromatin remodeling”, “vesicle transport” and “ergosterol biosynthetic process”. Because translation is related to various intracellular functions, vanillin may affect many intracellular processes indirectly. Several ribosomal protein deletion mutants (Δ*rpl21a*, Δ*rpl6b*, Δ*rps21a*, Δ*rpl37a*, and Δ*rpl12b*) exhibited vanillin-sensitive phenotypes [7]. Interestingly, none of the vanillin-sensitive mutants exhibited morphological phenotypes that were substantially similar to vanillin-treated cells, although two of them (Δ*rps21a* and Δ*rpl12b*) exhibited a small cell size [17], [37].

Further progress in understanding the effects of vanillin on yeast growth will require discovery of methods of repressing translational activity after treatment with vanillin, as well as elucidation of the mechanism of vanillin tolerance. Identification of the cellular components that bind to vanillin is also crucial.

## Supporting Information

Figure S1 **Vanillin concentration in culture of Δ** ***his3,*** **Δ** ***adh6*** **and Δ** ***adh7*** **cells.** Δ*his3* (open circles), Δ*adh6* (closed diamonds), and Δ*adh7* (closed circles) cells were pre-cultured in YPD and inoculated at a concentration of 2×10^6^ cells/ml into YPD containing 2.5 mM vanillin. Supernatants were collected at the indicated time point and vanillin concentrations were measured. Open squares indicate YPD without cells.
(EPS)

Figure S2 **Features of morphological changes of Δ** ***his3*** **(** ***ERG6*** **) and Δ** ***erg6*** **mutant cells after treatment with vanillin.** Δ*his3* and Δ*erg6* cells were grown in YPD medium with various concentration of vanillin, and the fluorescence images were analyzed as described for Figure 1. The experiments were repeated five times. Z values of Jonckheere–Terpstra test for each parameter were transformed into 102 principal component scores using null distributed data (n = 122, see Materials and Methods). The Pearson’s correlation coefficient of 102 PC scores between Δ*his3* and Δ*erg6* was 0.529 (*P*<5×10^−8^, two-sided *t*-test).
(EPS)

Figure S3 **The assembly of cytoplasmic processing bodies (P-bodies) after treatment with vanillin.** Assembly was confirmed using other components of P-bodies. Cells were treated with 0–50 mM vanillin for 30 min or deprived of glucose (- Glc) for 15 min.
(TIF)

Figure S4 **The assembly of stress granules (SGs) after treatment with vanillin.** Assembly was confirmed using other SG components. Cells were treated with 0–50 mM vanillin for 30 min, deprived of glucose (- Glc) for 15 min, or administered robust heat shock at 46°C for 10 min.
(TIF)

Table S1 **List of strains used in this study.**
(XLS)

Table S2 **List of parameters detected by Jonckheere-Terpstra test at P<0.01.**
(XLS)

Table S3 **List of strains having morphological profiles similar with that of the vanillin treated cells.**
(XLS)
