# Supplementary figures and images for: Vanillin Inhibits Translation and Induces Messenger Ribonucleoprotein (mRNP) Granule Formation in Saccharomyces cerevisiae: Application and Validation of High-Content, Image-Based Profiling
Source: PLoS One. 2013 Apr 24;8(4):e61748. doi: 10.1371/journal.pone.0061748 (PMC3634847; doi:10.1371/journal.pone.0061748)

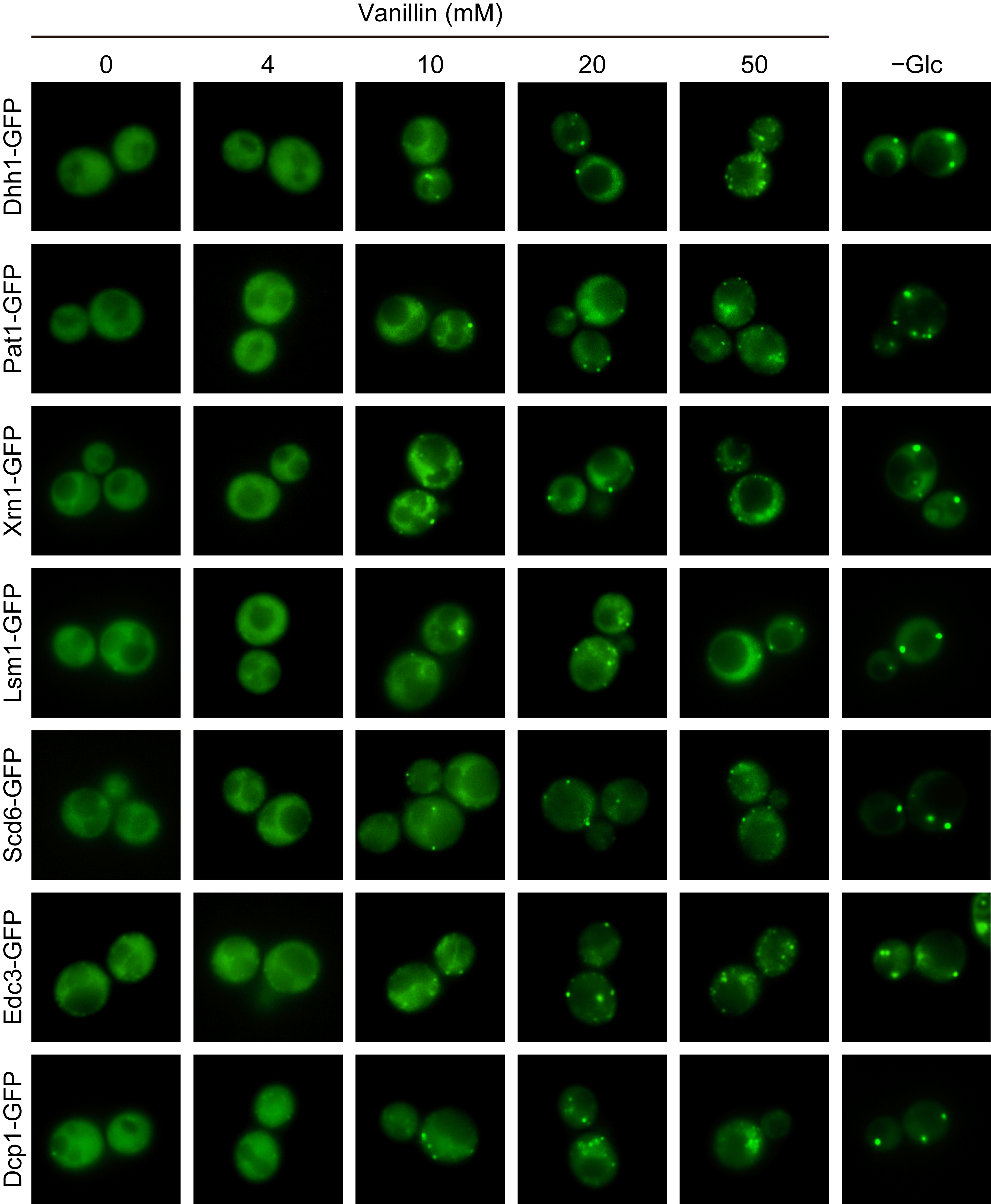

Supplement: Figure S3 — The assembly of cytoplasmic processing bodies (P-bodies) after treatment with vanillin. Assembly was confirmed using other components of P-bodies. Cells were treated with 0–50 mM vanillin for 30 min or deprived of glucose (- Glc) for 15 min. (TIF) [file pone.0061748.s003.tif]

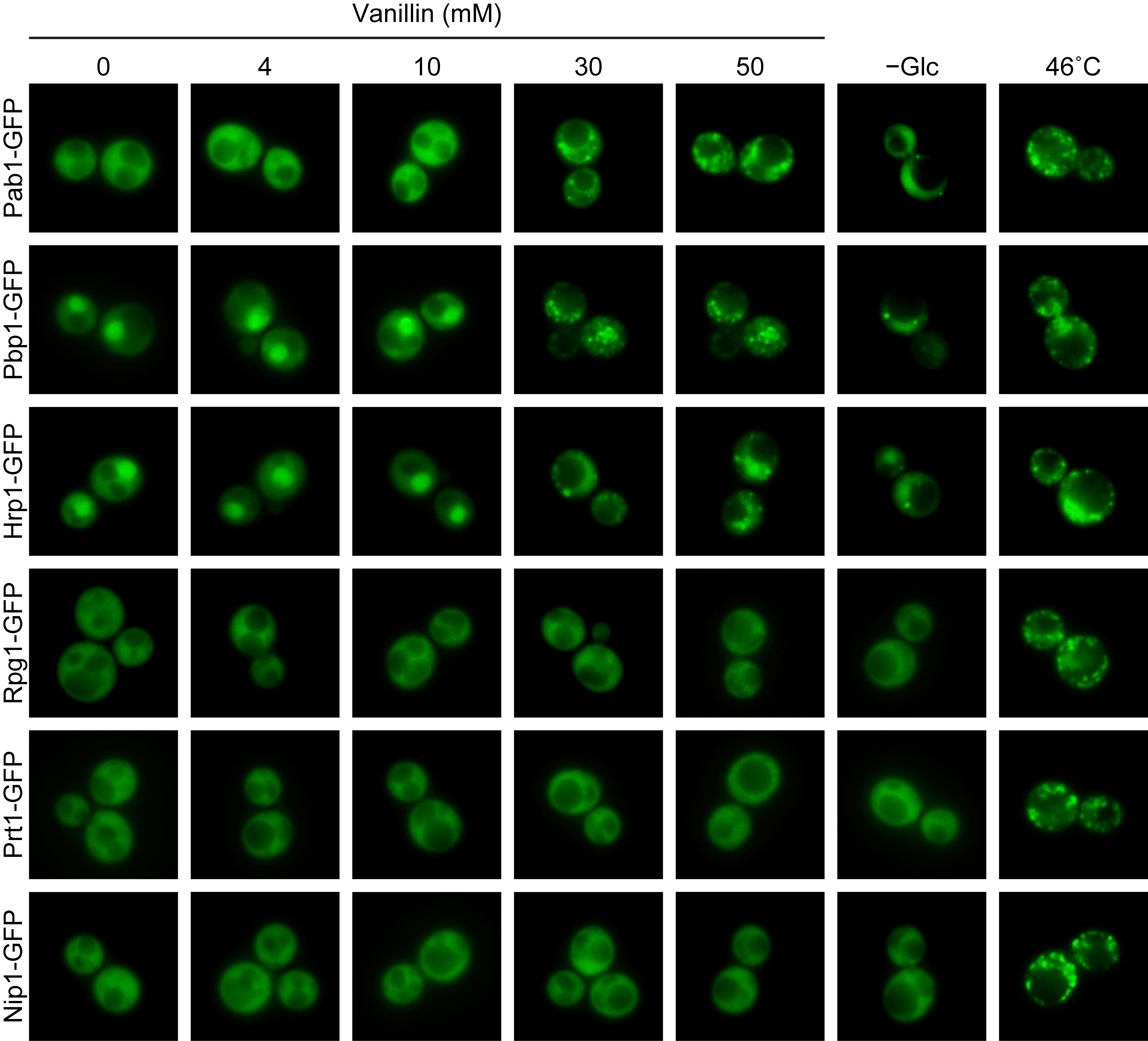

Supplement: Figure S4 — The assembly of stress granules (SGs) after treatment with vanillin. Assembly was confirmed using other SG components. Cells were treated with 0–50 mM vanillin for 30 min, deprived of glucose (- Glc) for 15 min, or administered robust heat shock at 46°C for 10 min. (TIF) [file pone.0061748.s004.tif]
